# Surgical Management of Hepatoblastoma and Recent Advances

**DOI:** 10.3390/cancers11121944

**Published:** 2019-12-04

**Authors:** Tianyou Yang, Richard S. Whitlock, Sanjeev A. Vasudevan

**Affiliations:** 1 Divisions of Pediatric Surgery and Surgical Research, Michael E. DeBakey Department of Surgery, Pediatric Surgical Oncology Laboratory, Texas Children’s Surgical Oncology Program, Texas Children’s Liver Tumor Program, Dan L. Duncan Cancer Center, Baylor College of Medicine, Houston, TX 77030, USA; mdtianyouyang@hotmail.com (T.Y.); richard.whitlock@bcm.edu (R.S.W.); 2 Department of Pediatric Surgery, Guangzhou Women and Children’s Medical Center, Guangzhou Medical University, Guangzhou 510623, Guangdong, China

**Keywords:** hepatoblastoma, liver resection, metastasectomy, children

## Abstract

Hepatoblastoma is the most common childhood liver malignancy. The management of hepatoblastoma requires multidisciplinary efforts. The five-year overall survival is approximately 80% in developed countries. Surgery remains the mainstay of treatment for hepatoblastoma, and meticulous techniques must be employed to ensure safe and effective local control surgeries. Additionally, there have been several advances from both pediatric and adult literature in the way liver tumor surgery is performed. In this review, we highlight important aspects of liver surgery for hepatoblastoma, the management of metastatic disease, and the most current technical advances in performing these procedures in a safe and effective manner.

## 1. Introduction

Hepatoblastoma is the most common liver malignancy in children. The incidence is approximately 1.2/1,000,000 and about 100 new cases are diagnosed annually in the United States [1]. Hepatoblastoma accounts for over 90% of the primary hepatic malignancies among children less than 5 years of age [1]. The incidence of hepatoblastoma has increased over the past two decades, partially due to the increased survival of premature and low-birth-weight infants [2,3,4,5]. Hepatoblastoma usually presents with a large abdominal mass and an elevated α-fetoprotein protein (AFP) value, mostly affecting children less than 3 years of age. The current mainstay treatment includes chemotherapy, surgical resection, and transplantation. The advent of platinum-based chemotherapy regimens has dramatically improved the outcomes of hepatoblastoma. Although the Children’s Oncology Group (COG), the International Childhood Liver Tumors Strategy Group (SIOPEL), the Society for Pediatric Oncology and Hematology (GPOH), and Japanese Pediatric Liver Tumors Group (JPLT) use different platinum-based chemotherapy regimens, the overall survival in each group is quite similar [6,7]. Surgery plays a critical role in the management of hepatoblastoma, and complete resection is the only way to achieve cure [8]. Surgical techniques and surgical tools have advanced in past decades which has greatly facilitated precision hepatectomies and metastasectomies. Furthermore, orthotopic liver transplantation provides promising outcomes for those with unresectable hepatoblastoma. The five-year overall survival rate for hepatoblastoma is approximately 80% with those who underwent partial hepatectomy achieving survival rates as high as 91% [9]. Upon review of 292 patients (in 29 separate publications) with hepatoblastoma who underwent liver transplantation, 76% of patients were alive at the time of publication. Forty-one percent of patients with rescue liver transplantation survived, compared with 85% of the patients with primary liver transplantation [10]. These achievements in hepatoblastoma treatment are the results of joint international efforts which have led to the development of treatment guidelines. Transplantation for hepatoblastoma has been recently reviewed [11]. In this review, we mainly focus on the surgical resection of hepatoblastoma. 

## 2. Preoperative Planning

A variety of radiographic tools can be used to image hepatoblastoma, and surgical resectability should be evaluated based on the combination of these imaging findings. Ultrasound is the preferred modality for the initial screening and diagnosis of an abdominal mass. Hepatoblastoma usually presents with a heterogeneous echo signal and significant mass effect on adjacent organs. Ultrasound can confirm the hepatic origin of the tumor by evaluating the movement of the mass with respiration or its vascular supply emanating from the portal vein and hepatic artery [12]. Ultrasound is also very sensitive for detecting portal vein, hepatic vein, and inferior vena cava thrombus [13]. 

Magnetic resonance imaging (MRI) is the preferred cross-sectional imaging modality for the primary tumor because it provides superior soft tissue contrast resolution and does not employ ionizing radiation [13]. On MRI, hepatoblastoma typically has a heterogeneous appearance with a hyperintense signal on T2-weighted images and a hypointense signal on T1-weighted images [13]. The development of hepatocyte-specific MRI contrast provides radiologists with a very useful tool in diagnosing liver tumors [14]. Gadolinium-based compounds are currently used for hepatocyte-specific MRI contrast media. Gadolinium-ethoxybenzyl-diethylenetriamine penta-acetic acid (Gd-EOB-DTPA) has been very useful in the evaluation of pediatric liver lesions, particularly in the sharp distinction of the tumor from normal liver parenchyma, the clear delineation of tumor margins with respect to the biliary tree and blood vessels, and the presence of satellite lesions not otherwise picked up by CT scan. This contrast agent has the potential to improve characterization and staging of hepatoblastoma [15]. Additionally, diffusion-weighted imaging is emerging as a useful sequence for liver lesion detection and characterization. Highly cellular and malignant lesions tend to demonstrate restricted diffusion on this sequence [16]. It can be very useful in detecting and confirming multifocal hepatoblastoma. Vascular invasion is best depicted with gradient-echo imaging or contrast enhanced MR angiography (MRA) [12]. Furthermore, MRA can also detect normal anatomic vascular variations that can guide surgical resection.

Since approximately 20% of the newly diagnosed hepatoblastomas present with lung metastasis [17], multi-detector computed tomography (MDCT) lung scanning is required at initial diagnosis and can also be used to scan the abdomen at the same time [13]. Tri-phase imaging is usually recommended for hepatoblastoma which consists of arterial, portal-venous, and hepatic-venous phases. The MDCT appearance of hepatoblastoma is highly variable and depends on the tumor’s histologic composition. Although MDCT has fallen from favor over the last decade because of the risks of radiation toxicity and lower detection rate and diagnostic accuracy compared to MRI [18], physicians can justify the use of this scan to reduce the need for anesthesia/sedation [13].

Positron emission tomography (PET)/CT has no definitive role in the diagnosis of hepatoblastoma. However, PET/CT can provide valuable information in the assessment of relapsed cases and is especially useful for detecting small early recurrences [19,20,21].

## 3. The PRETEXT and POST-TEXT System

The pre-treatment extent of tumor (PRETEXT) system was developed by SIOPEL to standardize imaging evaluation and risk stratification for hepatoblastoma prior to neoadjuvant chemotherapy [13,22,23,24], whereas the POST-TEXT (post-treatment extent of disease) system uses the same standards as PRETEXT (Table 1) but classifies hepatoblastoma during neoadjuvant chemotherapy [13,23].

In addition to these classifications, PRETEXT annotation factors, such as hepatic venous involvement, portal venous involvement, extrahepatic disease, multifocality, rupture, caudate involvement, lymph node metastases, and distant metastases, should also be carefully addressed based on MRI/MDCT findings, according the 2017 PRETEXT guideline [13]. The PRETEXT and POST-TEXT are powerful tools for predicting resectability and survival of hepatoblastoma patients. Detailed and accurate PRETEXT or POST-TEXT staging is of great importance in the surgical management of hepatoblastoma. However, PRETEXT is only approximately 50% accurate compared to pathology assessment with a tendency to over-stage patients using the PRETEXT system [24]. It is recommended that PRETEXT III and IV hepatoblastomas should undergo a central radiology review at diagnosis as well as before surgery.

The Children’s Hepatic Tumors International Collaboration-Hepatoblastoma Stratification (CHIC-HS) has been proposed for the newly launched Pediatric Hepatic International Tumor Trial (PHITT) which will prospectively validate this proposed model [9]. Based on the PRETEXT system, age, and AFP levels, CHIC-HS classifies hepatoblastoma into very low-risk, low-risk, intermediate-risk, and high-risk groups [9].

## 4. Upfront Versus Delayed Surgery

The International Childhood Liver Tumors Strategy Group and Children’s Oncology Group approach a newly diagnosed hepatoblastoma with different strategies. The International Childhood Liver Tumors Strategy Group tends to give neoadjuvant chemotherapy to all hepatoblastoma patients and then performs a delayed surgery [22,25,26]. The advantage of giving neoadjuvant chemotherapy to all hepatoblastoma patients is that size reduction and down staging of the tumor can be achieved in the majority of cases [27]. Advanced-stage tumors can greatly benefit from neoadjuvant chemotherapy. The SIOPEL trials have revealed that more than half of PRETEXT IV tumors can be completely resected with partial hepatectomy after intensified neoadjuvant chemotherapy [28]. Children’s Oncology Group favors up-front resection for initially resectable hepatoblastoma and gives neoadjuvant chemotherapy only to those deemed unresectable in hopes of reducing total chemotherapy administered [29]. For well-differentiated fetal hepatoblastoma, which can be cured with complete surgical resection alone, this approach avoids the need for chemotherapy [8]. Of note, the diagnosis of well-differentiated fetal hepatoblastoma can only be established based on a primary resected sample [30]. It is possible that some of the PRETEXT I and II patients who underwent primary resection may also benefit from reduced cycles of postoperative chemotherapy. Recent PHITT surgical guidelines recommend primary resection only for PRETEXT I and PRETEXT II patients; PRETEXT II tumors should have >1cm radiographic margin from the middle hepatic vein, the retro-hepatic inferior vena cava, and the portal bifurcation. Also, upfront trisectionectomy is no longer recommended for any newly diagnosed hepatoblastoma.

It is equally important to determine the optimal timing of delayed surgery for those who undergo neoadjuvant chemotherapy. The International Childhood Liver Tumors Strategy Group tends to give four cycles of neoadjuvant chemotherapy for standard risk hepatoblastoma [22,26]. Murphy et al. [31] demonstrated that hepatoblastoma volumes regressed significantly with increasing neoadjuvant chemotherapy cycles. However, tumors often remained anchored to the major hepatic vasculature, showing marginal improvement in resectability criteria. Another study found that after two cycles of neoadjuvant chemotherapy, the majority of stage III and IV hepatoblastomas either did or did not achieve radiographic resectability, and further chemotherapy did not change this outcome [32]. Tumor volume and serum AFP values can be used to assess the responsiveness of chemotherapy [33]. Generally speaking, tumor resectability should be reevaluated after every two cycles of neoadjuvant chemotherapy.

## 5. Advancement in Techniques to Make Tumors Resectable

Hepatoblastomas that are unresectable (PRETEXT IV, V+, P+) with standard liver resection should be referred for liver transplantation [34,35]. Early referral should be conducted in order to achieve the best possible outcome. Trans-catheter arterial chemo-embolization (TACE) alone, or in combination with high-intensity focused ultrasound, may be considered for those with unresectable tumors that are not responsive to primary systemic chemotherapy and are also not suitable for liver transplantations [36,37,38,39]. Furthermore, transarterial radioembolization with yttrium-90 could be considered as adjunctive therapy in unresectable hepatoblastoma and could be used as a bridge to surgical resection or liver transplant [40].

In the scenario of possible insufficient future liver remnant (FLR), the associating liver partition and portal vein ligation for staged hepatectomy (ALPPS) procedure will be of great usefulness for increasing the volume of FLR [41,42,43]. The ALPPS procedure is a two-staged liver resection, combining two established surgical techniques: portal vein ligation and in situ splitting of the liver. During the first stage, the liver is completely divided from the FLR with concomitant portal vein ligation of the lobe that will be removed while preserving ipsilateral arterial blood supply, bile duct, and hepatic vein drainage. The second stage is usually performed 1–2 weeks later with removal of the liver with its portal vein ligated [44,45]. Wiederkehr et al. [45] reported the initial experiences of ALPPS procedure performed in five pediatric patients; a rapid growth of the remnant livers was observed in all but one patient. The increase in the ratio of FLR to total liver volume ranged from 62% to 102% in four patients. The only postoperative complication was an asymptomatic right pleural effusion that was aspirated during the second stage procedure. The ALPPS has also been successfully performed in a 54 day old infant with hepatoblastoma [43]. However, since as little as 20–25% of FLR is sufficient for pediatric patients undergoing liver resection, ALPPS should only be reserved for those high-risk patients who would otherwise not be a liver transplant candidate. Additionally, portal vein embolization alone is another option to induce hypertrophy of FLR.

## 6. Advances Intraoperative Techniques and Approaches to Local Control

In recent years, new technological advances have been employed as adjuncts to pediatric hepatic resections with exciting results. These include the use of intraoperative ultrasound, the use of image guided three-dimensional reconstruction, and the use of indocyanine green (ICG) during liver resection and metastatic lesion resection. Newer approaches to local control surgery have been to use laparoscopic liver resection and more extreme resection techniques.

Intraoperative ultrasound (IOUS) during hepatic resection in adults was first described with good results as early as the 1970s. The use of IOUS as an adjunct to adult liver resections in cases of both hepatic metastasis from gastrointestinal malignancies as well as for defining the proper hepatic transection planes is well reported with good results [46]. The use of IOUS has continued to lead to changes in operative strategy despite well-reported advances in preoperative imaging. While the use of IOUS in pediatric liver resections has been reported as early as 1999, most reports were of only a few patients from single center institutions. In 2015, Felsted et al. [47] described a study examining the use of IOUS in pediatric patients undergoing liver resection and found that, even with the advances in preoperative MRI imaging, discordant findings were found in approximately 20% of patients intraoperatively with ultrasound that changed the operative management in 14% of cases. While it is established that MRI is the best choice for preoperative imaging of pediatric hepatic malignancies with sensitivity of 1–3 mm, there are cases in which MRI is limited. In cases of complex or distorted hepatic vascular anatomy, the use of IOUS can be used to better assess the relationship of the tumor to the corresponding hepatic veins. Of note, particularly challenging in preoperative imaging is that tumors that occupy segment IV can be distorted secondary to the umbilical fissure, unclear middle vein trajectory, and sometimes obliterated left portal vein secondary to tumor involvement [47]. The images of preoperative MRI might also further be distorted secondary to parenchymal compression from local tumor effects as well as motion artifacts during the time of imaging. With IOUS, a spatial resolution of 3–5 mm can be obtained. Most importantly, the tissue is able to be moved as needed in order to best visualize important structures and determine relationships in an effort to achieve a proper transection plane in a parenchyma sparing resection. Ultrasound should be readily available to the surgeon to ensure the correct plane of parenchymal transection and preservation of feeding and draining vessels to the remnant liver.

**Figure 1:**
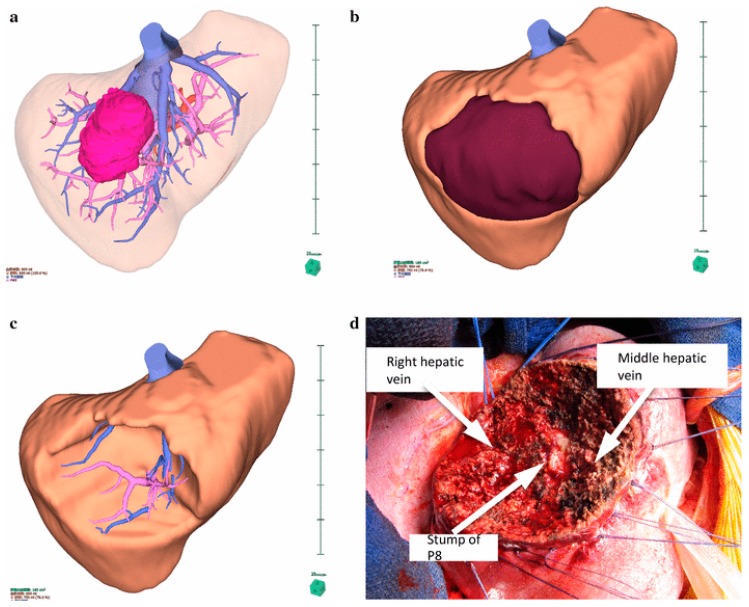
Virtual 3D simulation in a case of segment 8 segmentectomy (Adopted from [50]). The tumor is located in segment 8 of the liver (**a**). S8 segmentectomy was planned, and the resection line was drawn along the demarcation line of segment 8 portal vein (**b**). An image of virtual resection of segment 8 (**c**). The position of the stump of the segment 8 portal vein and the running directions of the middle hepatic vein and the right hepatic vein were similar to those seen in the preoperative simulation (**d**).

Technological advances have now created 3D image-processing software dedicated to virtual simulation in liver surgery (Figure 1). Such techniques provide not only 3D reconstructions of the liver structures, but also produce individualized anatomical information of the tumors and vascular structure. Surgeons can virtually perform liver resections under realistic anatomic conditions, evaluating the impact of the resection on the blood supply and the drainage of the liver remnant [48,49]. Three-dimensional tumor visualization and virtual simulation of tumor resections provides the basis to successfully plan for the treatment of a complex tumor.

Virtual resections can substantially contribute to surgical strategies for children with complex hepatic tumors [51]. This tool may also determine which specific surgical procedure is required such as an extended liver resection instead of primary transplantation in certain conditions [52]. Additionally, three-dimensional printing is taking a step forward by producing a real, tactile disease model. Three-dimensional printing models may help in planning surgical resection and even guide real-time resection [53,54]. These models provide a practical, hands-on tool that has a number of unique applications for surgical planning [54,55]. This model accurately mimics the liver structure and has been demonstrated to be very useful in adult liver resection and liver transplantation [55].

Another adjunct for pediatric hepatic resection is the use of ICG imaging. The use of preoperatively injected intravenous ICG and near infrared imaging equipment has emerged as a novel therapy in cases of solid organ malignancy and metastatic resections [56]. The use of ICG imaging emerges from its ability to be taken up by normally functioning hepatocytes and then undergo a period of post-injection hepatic washout. The degree of hepatic washout has been proposed as a mechanism for predicting post-operative liver dysfunction in adults [57]. Protein-bound ICG fluoresces at a specific wavelength that is able to be picked up by specially designed intraoperative imaging equipment using near infrared laser technology. Using this specialized equipment, which was initially reported for adult biliary surgery for better visualization to avoid ductal injury [58], sites of hepatic primary malignancy and metastatic locations are able to be detected with green fluorescence. In addition to the identification of small and difficult to palpate lymph nodes and metastasis, the use of ICG can also be used to identify small, superficially located hepatic tumors. This modality can be used to help achieve a negative resection status, safely remove tumors from closely underlying vascular structures, as well as assess the degree of any remaining malignancy. Because of these advances, ICG has started to gain popularity in oncologic surgery. Indocyanine green can be used for imaging both primary liver cancers and metastases. Healthy liver tissue clears ICG within a few hours, whereas liver tumor tissue retains ICG [59]. The current literature studying ICG in liver surgery uses selective portal vein and hepatic vein injections to highlight segmental anatomy as a guide for anatomical liver resection and for identifying small satellite lesions [59,60]. The use of ICG is well reported internationally in the resection of hepatoblastoma [61,62,63]. Generally, ICG (0.5–1 mg/kg) is intravenously injected 48–72 h prior to surgery to ensure hepatic clearance. This method aids in determining the resection line and identifying residual tumors [64].

Laparoscopic liver resection is becoming increasingly popular for adult liver resection. In experienced hands, laparoscopic liver resections are safe with acceptable morbidity and mortality for major hepatic resections [65]. Laparoscopic surgery provides patients with smaller incisions, less blood loss, and shorter hospital stays [66]. Laparoscopic ultrasound is crucial, and a thorough knowledge of both B-mode and Doppler ultrasonography is mandatory for accurate laparoscopic liver resections. Use of on-demand intermittent inflow occlusion and low central venous pressure anesthesia are very effective for limiting blood loss during laparoscopic liver resection. Advanced technologies, such as energy devices and staplers, are required for laparoscopic operations. The energy devices are effective for transecting the superficial liver parenchyma. For dissection of deeper parenchyma, prior identification and selective hemostasis of larger vessels by the use of combination of cavitron ultrasonic surgical aspirator (CUSA) and energy devices and staplers is recommended [67]. Some pediatric surgeons have performed laparoscopic liver resection for selective childhood liver tumors, including hepatoblastoma [68,69], but limited space in the abdominal cavity remains a major obstacle for childhood laparoscopic liver resection [70]. However, hepatoblastoma usually significantly shrinks after neoadjuvant chemotherapy and is usually not associated with hepatic cirrhosis. These may help to facilitate a laparoscopic liver resection. When considering laparoscopic resection of hepatoblastoma, appropriate tumor and patient selection is undoubtedly the key to success [70,71].

Extreme liver resection for advanced-stage (POST-TEXT III and IV) hepatoblastoma seems to have comparable overall survival when combined with chemotherapy as compared to liver transplantation [72,73]. Two major studies reporting these results presented patients with positive microscopic resection margins without local recurrence [72,73]. The application of intensive neoadjuvant chemotherapy regimens increases the possibility of extensive liver resection. In patients with an initial PRETEXT-IV tumor as the only high-risk feature, half of the tumor can be completely resected with partial hepatectomy after intensive neoadjuvant chemotherapy. Joerg Fuchs et al. [28] reported extensive liver resection in a series of 27 cases of POST-TEXT III and IV hepatoblastoma, with a five-year overall survival of 80.7%. El-Gendi A et al. [74] reported a three-year overall survival in 86.6% of a series of 15 cases of POST-TEXT III and IV hepatoblastoma patients who underwent extensive liver resection. Recent evidence suggested that, in the context of cisplatin-based chemotherapy, the presence of microscopically positive resection margin did not influence the outcome [75,76]. In the SIOPEL study, with a median follow-up of 67 months, local relapse occurred in 3/58 (5%) patients with microscopically positive resection margin and in 23/371 (6%) patients with complete resection. The 5 year overall and event-free survival was 91% and 86%, respectively, for the microscopically positive resection margin group and 92% and 85%, respectively, for the complete resection group [76]. This renders further support for extensive liver resection and may increase the chances of patients with POST-TEXT III and IV to undergo extensive liver resection. This aggressive surgical resection may mitigate the need for orthotopic liver transplantation in selective advanced cases. However, preparation for backup liver transplantation should always be considered.

## 7. Resection of Lung Metastasis

The lung is the most common metastatic site for hepatoblastoma and approximately 20% of the initially diagnosed hepatoblastoma cases have also presented with lung metastasis [28]. The initial treatment for hepatoblastoma with lung metastasis is neoadjuvant chemotherapy. Among the patients with initial lung metastases, more than half achieved complete remission of the lung lesions with intensive neoadjuvant chemotherapy [28,77,78]. Metastasectomy for residual pulmonary nodules after neoadjuvant chemotherapy should be aggressively performed [79]. Wanaguru et al. [78] reported that aggressive surgical resection of lung metastasis achieved long-term cure in eight hepatoblastoma cases in the context of chemotherapy. The only absolute contradictions for metastasectomy would be insufficient pulmonary function. Moreover, lung metastasis should always be resected prior to liver transplantation.

Lung metastasis can be safely resected with traditional thoracotomy or thoracoscopic surgery. The traditional thoracotomy provides the ability to manually palpate the lungs, which was once considered essential in identifying lung metastasis. For video-assisted thoracoscopic surgery, CT-guided lung nodule localization using the combined techniques of methylene blue blood patch and hook wire is safe, technically feasible, and successful in children. Using this combination of techniques will consistently yield a pathological diagnosis [80].

**Figure 2:**
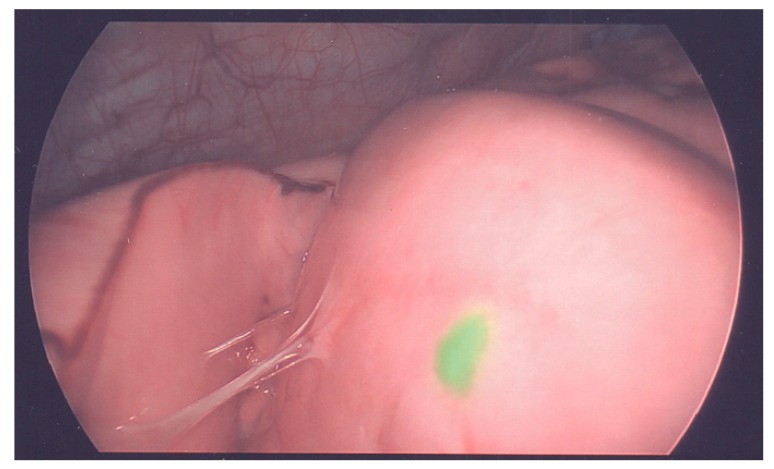
Indocyanine green (ICG) guided thoracoscopic surgery. Green color indicates lung metastasis.

Intraoperative ICG fluorescence imaging is feasible and useful for identifying small viable metastatic lung lesions (Figure 2). Kitagawa et al. [81] reported that ICG can detect lung lesions as small as 0.062 mm in diameter, and all of the pathologically positive lesions were clearly fluorescence positive in a study of 10 patients. Most researchers suggested an interval between the administration of ICG (0.5 mg/kg) and pediatric surgery of at least 1–4 days to decrease the background fluorescence [62,64,81]. Navigation surgery using ICG could not detect the tumors located at a depth of more than 10 mm from the organ surface [62].

## 8. Management of Disease Relapse

Relapsed hepatoblastoma occurs in less than 12% of patients with complete remission after the first line of treatment [82]. Most of the relapses happen in the liver and lungs. Combined treatment with chemotherapy and surgical removal is essential for long-term survival [82,83]. All four patients in the JPLT-1 study with local relapse were salvaged with chemotherapy and surgical resection [83]. In the SIOPEL series, 52% (31 of 59) of the relapsed patients achieved a second complete remission. Although surgical resection of a local relapse may be difficult after a previous surgery, complete surgical resection was achieved in 15 out of 21 patients who experienced local relapse in the SIOPEL study [82]. Indocyanine green may offer accurate guidance in the resection of the relapsed disease because it enables the identification of small viable lesions during surgery that otherwise may not be defined by cross sectional imaging [64,84,85]. It can be used not only for the guidance of a second resection for the local relapse but also for the clearance of any residual pulmonary nodules in preparation for orthotopic liver transplantation.

Orthotopic liver transplantation is an option for those with unresectable local relapse [85]. However, Otte et al. [86] reported that children who undergo rescue liver transplantation have a considerably worse prognosis compared with those who undergo primary liver transplantation. The six-year post-transplantation overall survival was 82% for 106 patients who received a primary orthotopic liver transplantation but only 30% for 41 patients who underwent a rescued orthotopic liver transplantation. A moderate increased success rate was reported by Trobaugh-Lotrario [10] through summarizing data in 29 separate publications. Of 41 patients with rescue liver transplantation after initial attempt at resection, seventeen (41%) were alive [10]. Encouragingly, Sakamoto et al. [87] reported a 78% of three-year recurrence-free survival in a cohort of 15 patients with a rescued orthotopic liver transplantation. Percutaneous ablation therapy and transarterial radioembolization using yttrium-90 microsphere might be an effective alternative for the control of unresectable local relapse in patients after liver transplantation [88,89].

Lung relapse can be solitary or multiple, unilateral or bilateral [82]. The value of pulmonary metastasectomy for lung relapse is not as well-established as the management of residual lesions after neoadjuvant chemotherapy. Meyers et al. [79] reported that only 4 of the 13 patients with lung relapse who underwent thoracotomy were long-term survivors. Of note, five of them only had a thoracotomy and biopsy. Later data suggests that surgery and combined chemotherapy should be offered to all patients with lung relapse. In a SIOPEL study of 59 cases relapsed after achieving complete remission, 31 patients (52%) achieved a second complete remission. Of the twenty-seven patients that had lung relapses, 15 could be resected to achieve a second remission [82]. Shi et al. [90] reported surgical experiences of 10 patients with lung relapse, one with bilateral lung metastasis. Eight were effectively treated with pulmonary metastasectomy, which provided long term survival. The other two succumbed to extrapulmonary metastasis. Multiple repeat thoracotomies can be performed to clear pulmonary recurrences as needed in order to increase the disease-free interval.

## 9. Conclusions

The PRETEXT system and platinum-based chemotherapy have laid out a foundation for the current management of hepatoblastoma. Substantial progress has been made in the surgical arena of hepatoblastoma. Other than the promising outcomes of transplantation, the most exciting part of the surgical management of hepatoblastoma may be the introduction of ICG-guided and 3-D model guided surgery. These new technologies have led to precise resection of hepatic tumors that may not otherwise be resectable and can be effective for the treatment of primary or recurrent lung metastases. Furthermore, ALLPS further pushes the envelope for the success of surgical resection by increasing the volume of potentially insufficient future liver remnants. All of these advancements will enable surgeons to provide better surgical outcomes for hepatoblastoma.

## Figures and Tables

**Table 1:** The pre-treatment extent of tumor (PRETEXT) stage system.

Stage	Definition
PRETEXT I	Three contiguous hepatic sections are free of tumor
PRETEXT II	One or two sections have tumor involvement, but two adjoining sections are tumor-free
PRETEXT III	Two or three sections have tumor involvement, but no two adjoining sections are tumor-free
PRETEXT IV	All four sections have tumor involvement
